# Avascular Necrosis Secondary to Traumatic Posterior Hip Dislocation

**DOI:** 10.31486/toj.25.0062

**Published:** 2026

**Authors:** Eden Schoofs, Michael Christopher Marshall, Jonathan Willard, Deryk Jones

**Affiliations:** ^1^ Department of Emergency Medicine, Ochsner Clinic Foundation, New Orleans, LA; ^2^ Ochsner Andrews Sports Medicine Institute, Ochsner Clinic Foundation, New Orleans, LA; ^3^ The University of Queensland Medical School, Ochsner Clinical School, New Orleans, LA

**Keywords:** *Allografts*, *femur head necrosis*, *hip dislocation*, *hip injuries*, *osteoarthritis*, *osteochondritis dissecans*, *osteonecrosis*

## Abstract

**Background:**

Avascular necrosis of the femoral head, a known long-term complication following traumatic posterior hip dislocation, can result in femoral head collapse, osteochondral defects, and eventual posttraumatic osteoarthritis. This condition is especially problematic in young, active patients for whom total hip arthroplasty survivorship is a concern. Biologic treatments are therefore preferable to delay or avoid total hip arthroplasty.

**Case Report:**

A 16-year-old male developed progressive left hip pain after a posterior dislocation sustained while playing football. The hip was emergently reduced. Imaging (x-ray, computed tomography, and magnetic resonance imaging [MRI]) showed a small depressed cortical fracture on the anterior and superior femoral head. Conservative treatment included protected weight-bearing for 6 weeks, knee immobilization to restrict hip flexion, physical therapy, and a home exercise program. However, symptoms worsened after 5 months of conservative care. Repeat MRI demonstrated avascular necrosis with associated cartilage loss, hip effusion, and femoroacetabular impingement (a cam deformity). Given the patient's young age, persistent symptoms, functional limitations, and radiographic findings, the decision was made to perform surgical intervention. Surgical hip dislocation via an anterolateral approach with a small trochanteric osteotomy (modified Hardinge approach) was used to expose the femoral head while maintaining the remaining blood supply. Fresh femoral head osteochondral allograft transplantation was performed, along with femoral neck osteoplasty to address the cam deformity.

**Conclusion:**

Osteochondral allograft transplantation is an effective technique to treat osteochondral defects of the femoral head in the setting of avascular necrosis following traumatic hip dislocation. This surgical option restores hip function and may delay or prevent total hip arthroplasty in young, active patients.

## INTRODUCTION

Posterior native hip dislocation occurs when the femoral bone is forcefully internally rotated in an adducted position at the hip level, causing the femoral head to be displaced posteriorly relative to the acetabulum.^1^ Reports of anterior and anterior-inferior hip dislocations are rare.^2,3^ Posterior femoral head dislocation typically follows the high-energy trauma associated with motor vehicle collisions^4-7^ and less commonly occurs in athletes engaged in sporting activities such as football, snowboarding, skiing, and basketball.^8-10^ Native hip dislocation is an orthopedic emergency, as delayed reduction substantially increases the risk of avascular necrosis, posttraumatic osteoarthritis, and heterotopic ossification.^11^ Hougaard and Thomsen demonstrated that a hip reduction delay >6 hours substantially increased the likelihood of avascular necrosis.^12^ A meta-analysis performed by Ahmed et al also showed that earlier hip reduction (<6 hours from the time of injury) reduced the rate of avascular necrosis.^13^

When a patient has avascular necrosis of the femoral head, blood supply is lost and the bony architecture is weakened which can lead to femoral head collapse and progress to osteoarthritis. In young patients, the most common reasons for undergoing total hip arthroplasty (THA) are avascular necrosis and osteoarthritis.^14^

Osteochondral defects of the femoral head, although rare, can occur as a result of high-energy trauma, femoroacetabular impingement, osteochondritis dissecans, and other pathologies.^15^ These defects involve focal damage to both the articular cartilage and subchondral bone, thereby impairing joint function; causing synovitis, joint effusions, and mechanical symptoms; and predisposing the patient to degenerative changes.^15^

Because of its avascular nature, articular cartilage has poor intrinsic regenerative capacity, and damage to articular cartilage initiates an inflammatory cascade that can lead to progressive joint degeneration.^16,17^ For young patients with symptomatic osteochondral defects, early intervention is crucial to preserving hip function and delaying or preventing THA.^18^

Chondroplasty, microfracture, cartilage transplants, and other surgical techniques have been used to treat osteochondral injuries to the femoral head and other joint surfaces.^15,18^ However, because treatment for osteochondral defects has not been standardized, several treatment algorithms have been developed. These algorithms take into account that treatment options can be influenced by patient symptomology, etiology, lesion size, and activity goals.^19^ To guide treatment options, the International Cartilage Repair Society (now known as the International Cartilage Regeneration & Joint Preservation Society [ICRS]) developed a classification system based on the depth of the cartilage defect and macroscopic evaluation.^20^ Joint preservation techniques include osteochondral autograft transplantation, osteochondral allograft transplantation, and autologous chondrocyte implantation.^15,18,21^ Osteochondral allograft transplantation offers the advantage of restoring both the subchondral bone and articular cartilage in a single procedure, thereby providing a viable option for young, active patients with focal defects.^22-24^

We present the case of a young patient with an ICRS 4B lesion and underlying avascular necrosis secondary to traumatic posterior hip dislocation who underwent femoral head osteochondral allograft transplantation.

## CASE REPORT

A 16-year-old male presented to an outside hospital with left hip pain following a posterior hip dislocation sustained while playing football. The hip was reduced emergently, and postreduction pelvic radiographs demonstrated appropriate reduction. Postreduction magnetic resonance imaging (MRI) revealed a small depressed cortical fracture of the anterosuperior femoral head, subchondral bone edema, a small joint effusion, and extensive myositis involving the adductor and gluteal muscle groups. Computed tomography (CT) scan showed a well-reduced femoral head with an anterosuperior lesion (2.6-cm diameter and 2.6-cm depth). These studies were performed at an outside hospital and were not available for this report.

The patient was treated with protected weight-bearing, physical therapy, and a home exercise program. Because of worsening pain and stiffness, he discontinued physical therapy after 5 months of treatment.

The patient presented to our institution 6 months after his initial posterior hip dislocation. At the time of presentation, his walking distance was limited to 6 blocks, and he had difficulty putting on his socks and shoes. Preoperative patient-reported outcome measures were collected using validated methods. His left hip Harris Hip Score was 47.25 compared to a right hip score of 100. The Harris Hip Score uses both patient answers and physical examination to assess hip functionality and pain on a 100-point scale. Higher scores indicate less hip symptomology and better hip function.

The Western Ontario and McMaster Universities Osteoarthritis Index (WOMAC) is a self-reported questionnaire measuring pain, function, and stiffness on a 100-point scale, with higher scores indicating worse symptoms and greater limitation of the hip. The patient's WOMAC scores for the left hip were 45 for pain, 37.5 for stiffness, and 42.65 for function compared to right hip WOMAC scores of zero for all 3 measures.

We used the physical component score (PSF-12) and the mental component score (MSF-12) of the Short Form Health Survey to assess the patient's quality of life in terms of physical and mental health. The self-reported questionnaire has a maximum score of 100, and greater scores indicate better quality of life. Preoperatively, the patient's PSF-12 score was 17.85 and his MSF-12 score was 57.38.

**Figure 1.**
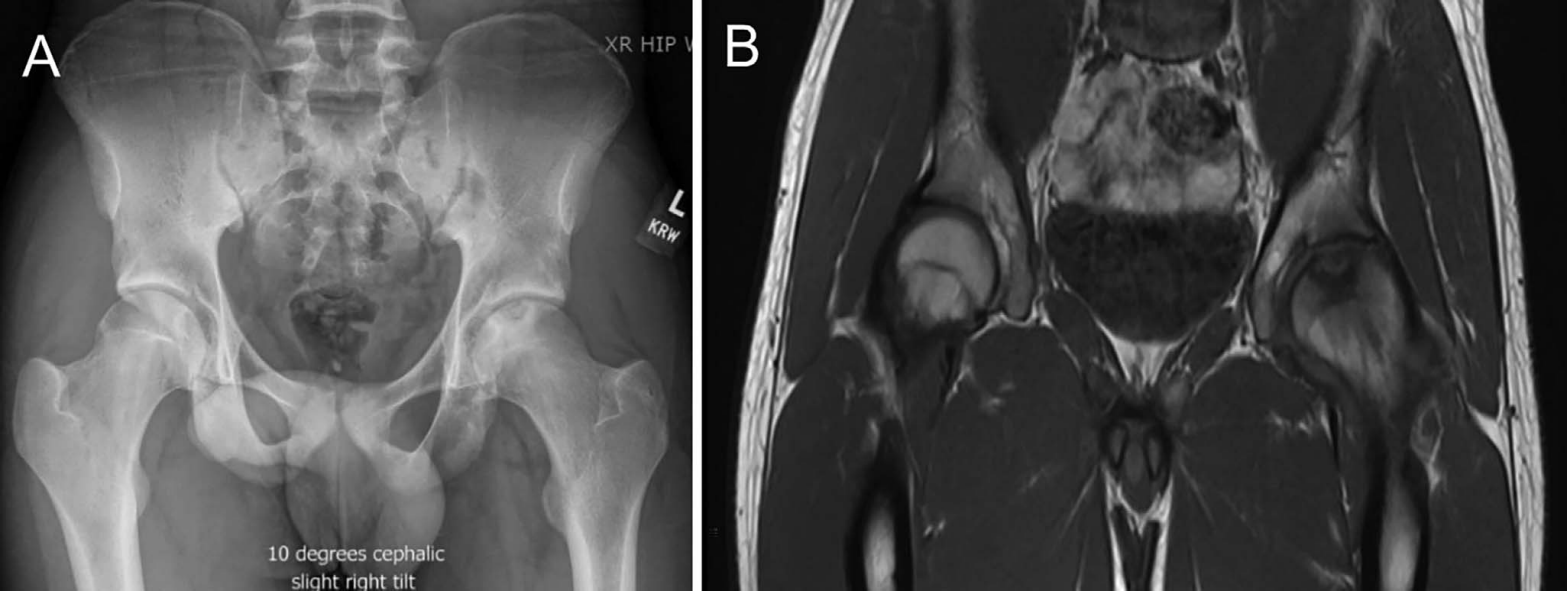
Preoperative imaging demonstrates hip pathology. (A) Pelvic 10° cephalic tilt bilateral hip radiograph shows a 1.5-cm left superior femoral head osteochondral defect and irregularity of the left humeral head. (B) Coronal T2-weighted, fat suppression bilateral hip magnetic resonance image shows left femoral head serpiginous double-line sign, indicative of avascular necrosis, cortical collapse, labral tear, subchondral edema, and hip effusion.

Because the initial radiographic studies were not available and considerable time had elapsed from the patient's hip dislocation, imaging studies were obtained to guide treatment options. Pelvic views revealed a 1.5-cm osteochondral defect of the left superior femoral head with mild anterosuperior joint space narrowing (Figure 1A). The appearance of the right hip was normal. MRI revealed a 1.6 × 2.8-cm anterosuperior lesion of the left femoral head with a serpiginous double-line sign consistent with avascular necrosis (Figure 1B), mild associated cortical collapse, and concomitant articular cartilage loss. The acetabulum demonstrated thinning with labral tearing as well.

Physical examination of the left hip revealed 10° abduction, 5° adduction, 60° flexion, and 0° internal rotation. The patient experienced pain with forced internal rotation when performing the flexion, adduction, internal rotation test and experienced pain with forced external rotation when performing the flexion, abduction, external rotation test. Circumduction, Stinchfield, and log roll tests were all positive.

Because of the patient's young age, desire to return to high-intensity activities, persistent symptoms, and radiographic findings, the decision was made to perform surgical intervention.

### Surgical Procedure

**Figure 2.**
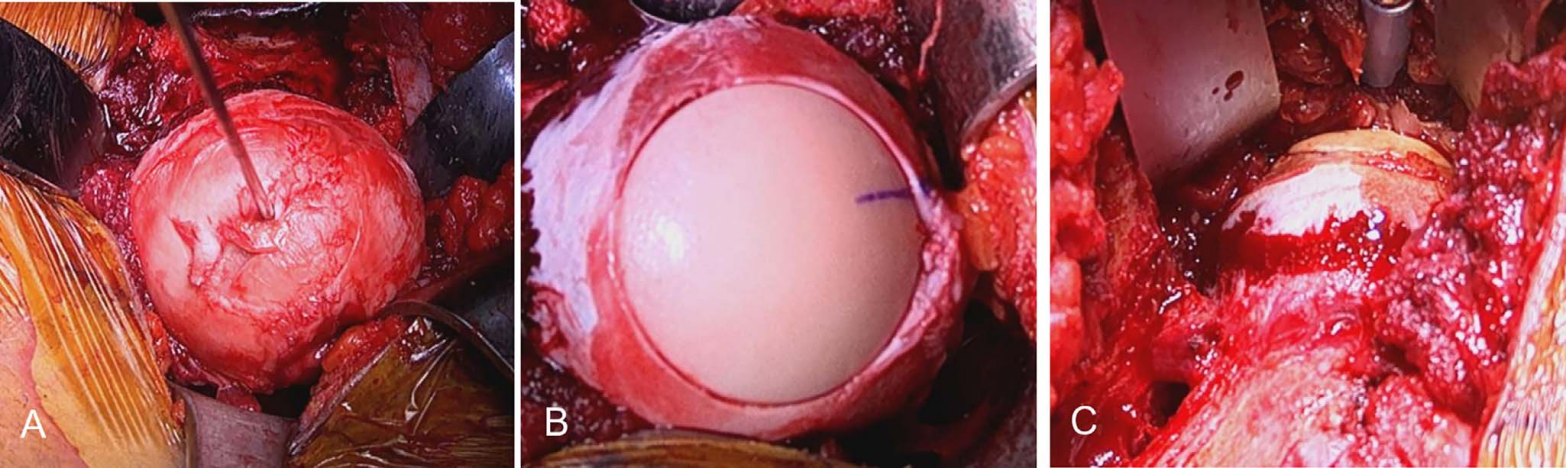
Intraoperative surgical pictures show osteochondral lesion, surgical hip dislocation, and graft transplantation. (A) The femoral head was prepared with a centralization pin based on the location and size of lesion. Note the cam lesion along the periphery of the native femoral head. (B) An osteochondral allograft was impacted into position and demonstrated excellent contour and fit. (C) The cam deformity was resected to prevent postoperative femoroacetabular impingement and maintain normal femoral head contour.

Surgical hip dislocation via a modified Hardinge approach was performed with a minimal trochanteric osteotomy.^25,26^ The surgeon noted a 3.5-cm ICRS 4B osteochondral lesion of the femoral head with a cam lesion, and following pin localization, the site was prepared to a depth of 0.8 cm (Figure 2A). An osteochondral allograft (MTF Biologics) preserved at 4 °C was cut to a diameter of 3.5 cm × a thickness of 0.8 cm, and the bone base of the graft was treated with carbon dioxide gas (CarboJet, Kinamed Inc) to remove the antigenic component from the donor graft. The graft was impacted into position with excellent fit and contour (Figure 2B). Femoral neck osteoplasty was performed to remove the cam lesion and ensure no femoroacetabular impingement; the hip was well reduced on completion of graft placement (Figure 2C). The labral tissues were intact, with minimal fraying noted, and required no repair or debridement. The minimal trochanteric osteotomy was repaired with transosseous suture fixation using #2 Orthocord suture (DePuy Synthes) placed through three 0.15-cm drill holes. The gluteus medius, gluteus minimus, and fascia lata were closed with #1 Vicryl (Depuy Synthes) figure-of-8 sutures. Standard deep space, subcuticular, and skin closures were performed.

### Postoperative Course

**Figure 3.**
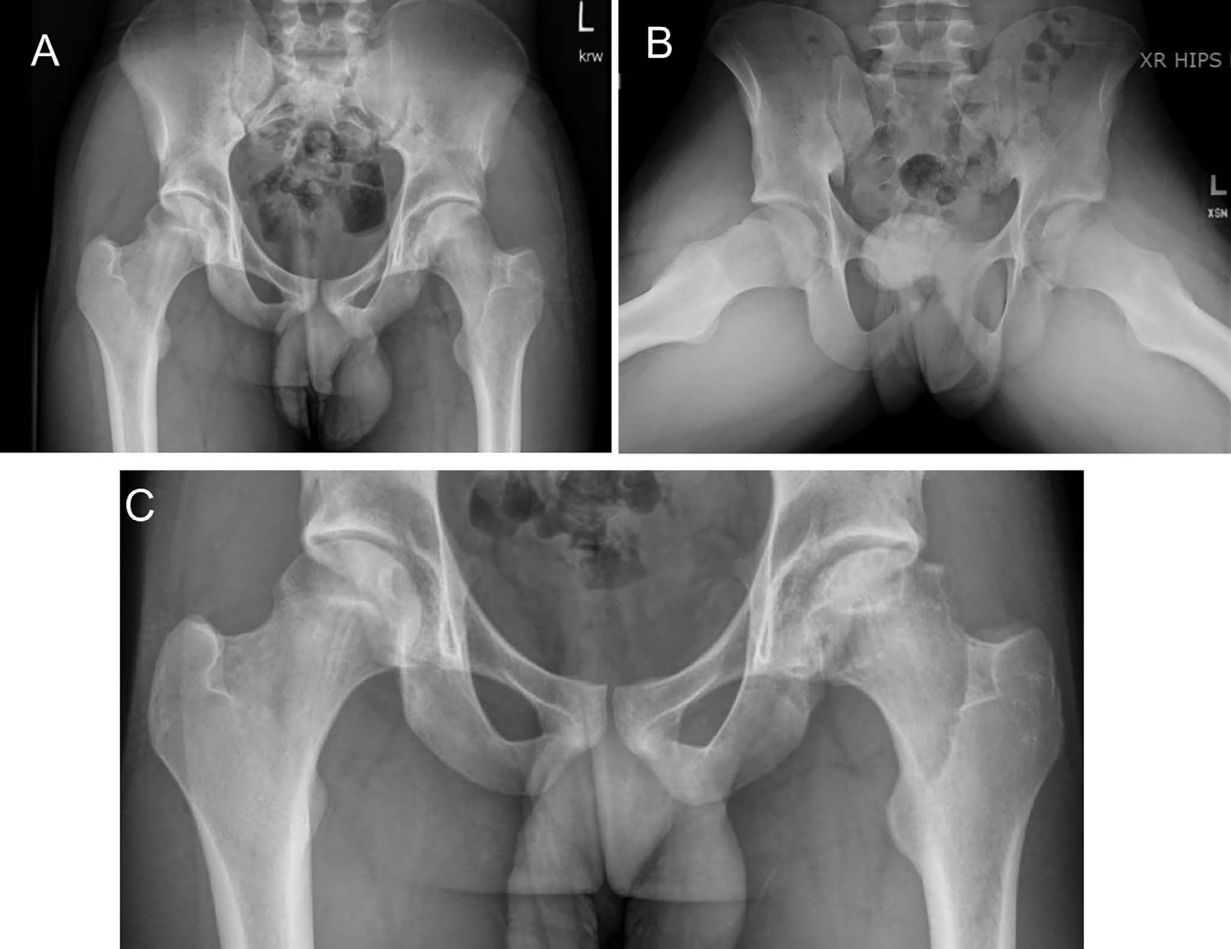
(A) Bilateral pelvic anterior-posterior radiograph 6 weeks postoperatively shows intact left hip osteochondral allograft with maintained joint space. (B) Bilateral frog lateral radiograph 3 months postoperatively shows excellent position of the left femoral head osteochondral allograft with no signs of collapse. (C) Bilateral pelvic anterior-posterior radiograph 24 months postoperatively shows minimal sclerosis in the left hip with small lateral femoral head bone spur formation, maintained left hip joint space, and intact left hip osteochondral allograft. Continued but diminished lucency is seen at the base of the left femoral head osteochondral allograft graft.

The patient was limited to toe-touch to 25% weight-bearing for 6 weeks, and range of motion (ROM) was limited to 0° to 60° with the use of a hip brace (Breg, Inc). Full ROM was allowed at 4 weeks, and full weight-bearing was allowed at 6 weeks. Postoperative radiographs at 6 weeks showed a well-reduced left femoral head with normal contour and the allograft in excellent position (Figure 3A). Six weeks postoperatively, the patient's left hip flexion was 90°, internal rotation was 5°, and external rotation was 0° with full extension. Motor strength was 5/5 for left hip flexion, abduction, and adduction. Anti-gravity treadmill (AlterG) rehabilitation was initiated at 6 weeks, along with a core strengthening program and progressive ROM. Hip abduction was limited for 3 months postoperatively and then progressed as tolerated.

At 3 months, the patient's gait and ROM had improved, left hip external rotation had improved to 30°, and he maintained left hip flexion of 90° and internal rotation of 5°. Muscular strength assessment demonstrated no limitations, consistent with the 6-week physical examination. Postoperative x-rays at 3 months showed excellent graft position and alignment of the left femoral head with appropriate integration (Figure 3B).

**Figure 4.**
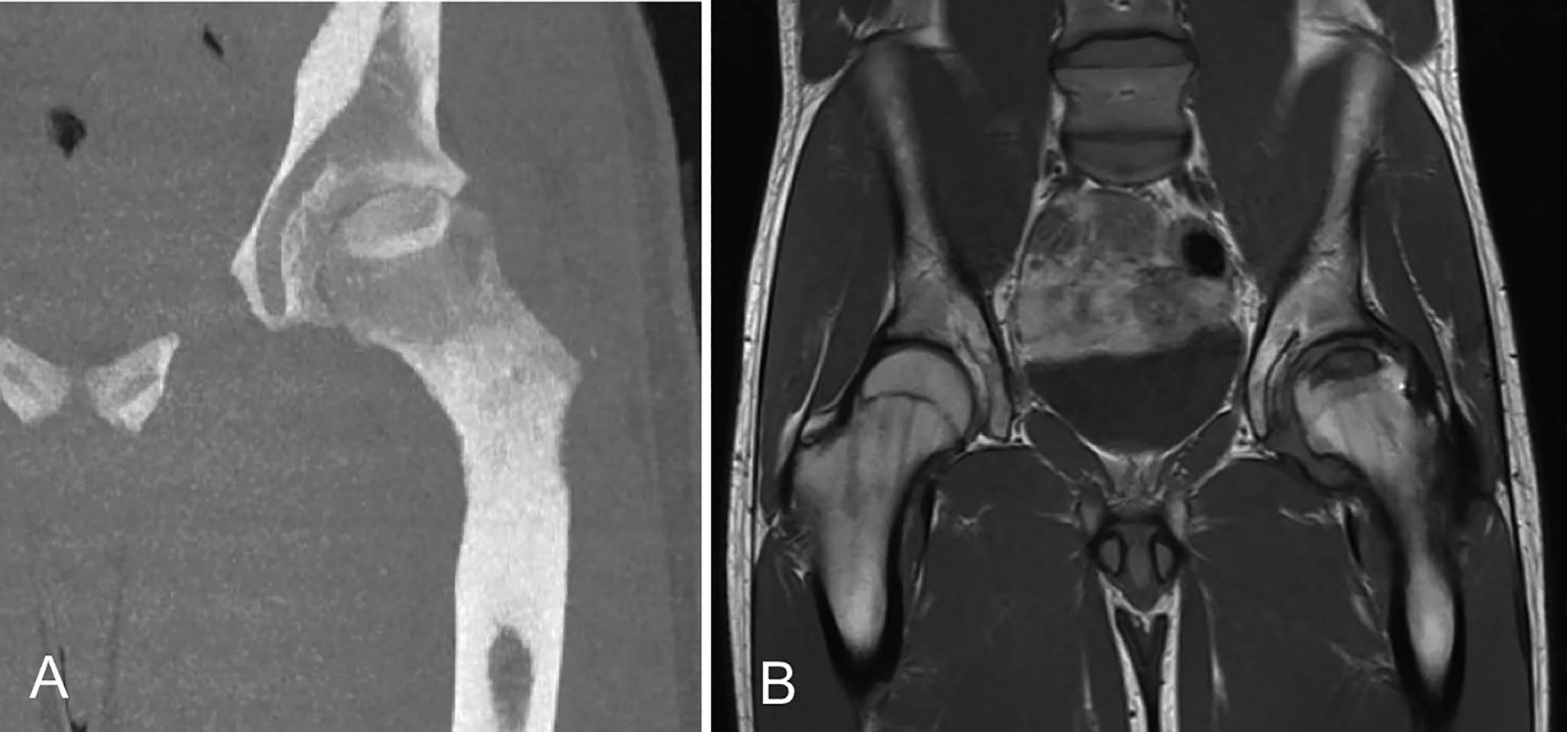
(A) Computed tomography scan at 24 months postoperatively shows maintained contour and alignment, demonstrated sclerosis, and no collapse. (B) Bilateral hip T1 magnetic resonance imaging shows a maintained bone and articular cartilage structure with poor vascularity of the graft.

At 24-month follow-up, the patient had graduated from high school and was working full time in a job that required constant movement and frequent lifting of >100 pounds. Despite the physical demands of his work, he reported no specific pain or symptoms. Improved ROM was noted with flexion of 100° and external rotation of 45°, as well as maintained abduction of 30°, extension of 0°, and internal rotation of 5°. Bilateral hip radiographic series and repeat CT studies demonstrated that the left femoral head graft was in excellent position with no collapse or significant asymmetric joint space narrowing, but some evidence of potential bridging with sclerosis of the graft was noted (Figure 3C and Figure 4A). MRI studies demonstrated maintained articular cartilage but continued avascularity of the graft (Figure 4B).

**Figure 5.**
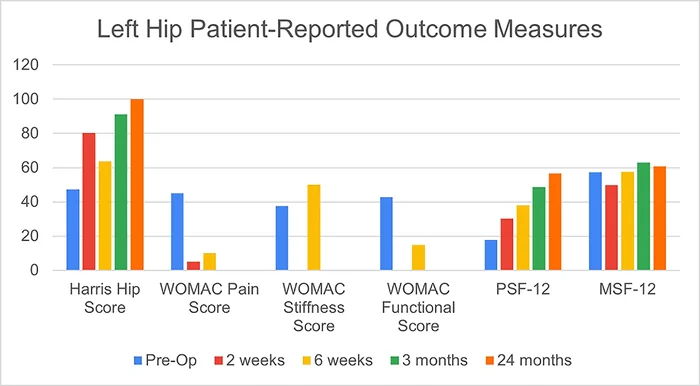
Patient-reported outcome measures scores from preoperatively (Pre-Op) to 24 months postoperatively. For the Harris Hip Score, the physical component score (PSF-12) of the Short Form Health Survey, and the mental component score (MSF-12) of the Short Form Health Survey, higher scores indicate better outcomes. For the Western Ontario and McMaster Universities Osteoarthritis Index (WOMAC) scores, lower scores indicate better outcomes. Timepoints without WOMAC scores signify a score of zero for the outcome measure.

Patient-reported outcome measures scores demonstrated progressive improvement from baseline to 24 months. The Harris Hip Score increased by 52.75 points. WOMAC scores decreased, with a 45-point decrease in the pain score, a 42.65-point decrease in the function score, and a 37.5-point decrease in the stiffness score, all signifying improvement. The PSF-12 improved by 38.73 points, and the MSF-12 improved by 3.38 points (Table and Figure 5).

**Table.** Patient-Reported Outcome Measures

		Western Ontario and McMaster Universities Osteoarthritis Index^b^			12-Item Short Form Health Survey^c^	
Time Point	Harris Hip Score^a^	Pain	Stiffness	Function	PSF-12	MSF-12
Preoperatively (baseline)	47.25	45	37.5	42.65	17.85	57.38
2 weeks postoperatively	80.22	5	0	0	30.28	49.88
6 weeks postoperatively	63.74	10	50	14.71	38.04	57.61
3 months postoperatively	91.21	0	0	0	48.72	63.03
24 months postoperatively	100	0	0	0	56.58	60.76

^a^The Harris Hip Score is a 100-point scale that uses both patient answers and physical examination to assess the patient's hip functionality and pain. A greater score indicates less hip symptomology and better hip function.

^b^The Western Ontario and McMaster Universities Osteoarthritis Index (WOMAC) is a self-reported questionnaire that measures pain, function, and stiffness. Higher scores on the 100-point scale indicate worse symptoms and greater limitations.

^c^The PSF-12 (physical health) and MSF-12 (mental health) are assessments from the 12-Item Short Form Health Survey and are scored on a scale of 0 to 100, with 100 representing normal function.

## DISCUSSION

In this case, 2 of the most serious consequences following femoral head dislocation occurred: an initial osteochondral defect of the femoral head was seen following reduction and despite emergent reduction, avascular necrosis developed. In a young, athletic patient, this catastrophic combination can lead to progressive femoral head collapse, osteoarthritis, and, traditionally, later THA. Despite advances in THA hardware, in young, active patients, THA can lead to accelerated polyethylene wear, and hardware loosening can cause joint failure and require revision.^27^ Other surgical techniques such as microfracture, osteochondral autograft transplantation, and osteochondral allograft transplantation can be used to treat osteochondral lesions in lieu of arthroplasty. El Bitar et al developed an algorithm for the treatment of hip chondral defects that is predominantly based on lesion size.^18^

Studies have shown that microfracture is favorable to arthroplasty and potentially limits lesion progression.^28,29^ Domb et al evaluated acetabular microfracture in 43 hips in 42 patients available for follow-up at 2 and 5 years.^28^ The 5-year results demonstrated 72.1% graft survivorship and no deterioration in patient-reported outcome measures, the visual analog scale assessing pain, or patient satisfaction.^28^

Good to excellent results have been reported with osteochondral autologous transfer from the knee or hip to the hip lesion.^21,30,31^ Autologous chondrocyte implantation has been reported by arthroscopically delivering either Novocart Inject (Octane Biotherapeutics Inc), which is biopsied cells from the femoral head, or Chondrosphere (ReLive Biotechnologies Ltd), which uses autologous blood to create cylinders.^30,31^ Thier et al evaluated 29 patients with acetabular lesions (average size 2.21 cm^2^) who were treated with either Novocart Inject or Chondrosphere self-adherent autologous chondrocyte implants. At 19-month postoperative follow-up, statistically significant improvement was seen in all patient-reported outcome measures (International Hip Outcome Tool, Euro-Quol group score, and Non-Arthritic Hip Score), with greater improvement seen in the Novocart Inject group.^30^

Osteochondral allograft transplantation is the treatment of choice for larger, deeper lesions, particularly of the femoral head in the setting of avascular necrosis.^18,32,33^ In a retrospective study by Oladeji et al, osteochondral allograft transplantation was used to treat femoral and acetabular lesions in 10 patients, and the authors identified avascular necrosis, smoking, acetabular involvement, and concomitant procedures as risk factors for conversion to THA.^34^ In the Oladeji et al study, 30% of patients underwent THA from 5 to 29 months after osteochondral allograft transplantation.^34^ In an evaluation of 8 patients treated with fresh femoral head osteochondral allograft transplantation, Kosashvili et al reported 87.5% graft survivorship at an average follow-up of 41 months, with 1 patient converted to THA and another patient requiring revision osteochondral allograft transplantation.^35^ In a retrospective review of 29 patients with femoral head lesions treated with osteochondral allograft transplantation, follow-up ranged from 0.6 to 13.7 years (average 6.6 years), and overall graft survivorship was 78.4% at 5 years and 62.7% at 10 years.^22^ A significant difference in graft survival was noted between patients with avascular necrosis (41.8% 10-year survival) vs patients treated for traumatic or osteochondritis dissecans (85.7% 10-year survival).^22^

Our patient's avascular necrosis placed him at risk for graft failure. For this patient, the senior author limited the bone load to 0.8 cm and preserved and verified healthy bleeding bone. The 2 most important issues in graft survivorship have historically been cell viability and bone incorporation.^36-39^ Our patient demonstrated graft survival and excellent patient-reported outcome measures 24 months postoperatively (30 months after initial posterior hip dislocation reduction). He was asymptomatic despite working a physically demanding job, and x-rays and CT scans demonstrated stable graft position with some evidence of bridging bone.

However, medium- and long-term survival of the graft may be impacted by the lack of graft incorporation demonstrated by MRI (Figure 4B). The persistent avascularity of the graft will require ongoing monitoring.

Limited data are available on the long-term complications of osteochondral allograft transplantation of the hip, but numerous studies report the medium- to long-term complications of osteochondral allograft transplantation of the knee. A systematic review of knee osteochondral allograft transplantations by Chahal et al demonstrated an 18% graft failure rate at a mean follow-up of 58 months.^40^ A systematic review of knee osteochondral allograft transplantations by Assenmacher et al reported a 25% graft failure rate and a reoperation rate of 36% after 12.3 years.^41^ Consequently, our patient should have yearly radiographs and clinical evaluations during the next 5 to 10 years.

In a review of the literature, we found limited data comparing osteochondral allograft transplantation with other surgical techniques for the femoral head, and even more limited data in the setting of avascular necrosis. However, from studies that compare osteochondral allograft transplantation to other joint-preserving techniques in the knee, we can extrapolate the knee findings and apply them cautiously to the hip. Krych et al conducted a meta-analysis comparing the rate of return to sport for microfracture, osteochondral autograft transplantation, osteochondral allograft transplantation, and autologous chondrocyte implantation used to treat osteochondral lesions in the knee.^42^ At a minimum 2-year follow-up, they found that overall, 76% of patients returned to sport following an osteochondral defect repair. Osteochondral autograft transplantation had the highest return to sport rate (93%), followed by osteochondral allograft transplantation (88%), autologous chondrocyte implantation (82%), and microfracture (58%). Osteochondral autograft transplantation also had the quickest recovery time at 5.2 months, while microfracture, osteochondral allograft transplantation, and autologous chondrocyte implantation took longer, averaging 9.1, 9.6, and 11.8 months, respectively.^42^ The Burroughs et al study of 2,598 patients with 5 to 10 years of follow-up after osteochondral defect repair demonstrated no significant difference in the rate of knee revisions between the osteochondral allograft transplantation and osteochondral autograft transplantation cohorts.^43^

Studies comparing joint-preserving techniques in the hip would be beneficial for assessing the efficacy of the various techniques.

## CONCLUSION

Osteochondral allograft transplantation for a traumatic femoral head lesion in the setting of underlying avascular necrosis can restore joint function, decrease pain, and allow resumption of demanding labor in a young, active patient. This case highlights successful surgery using a modified Hardinge approach, minimizing further compromise to femoral head vascular support, and transplanting a fresh osteochondral allograft femoral head. Two years postoperatively, the patient was doing well, but he will require medium- and long-term follow-up because avascular necrosis is associated with the risk of requiring subsequent THA.

## Notes

*This article meets the Accreditation Council for Graduate Medical Education and the American Board of Medical Specialties Maintenance of Certification competencies for Patient Care and Medical Knowledge.*
